# SCARLET (Supplemental Citicoline Administration to Reduce Lung injury Efficacy Trial): study protocol for a single-site, double-blinded, placebo-controlled, and randomized Phase 1/2 trial of i.v. citicoline (CDP-choline) in hospitalized SARS CoV-2-infected patients with hypoxemic acute respiratory failure

**DOI:** 10.1186/s13063-024-08155-0

**Published:** 2024-05-18

**Authors:** Sonal Pannu, Matthew C. Exline, Joseph S. Bednash, Joshua A. Englert, Philip Diaz, Amy Bartlett, Guy Brock, Qing Wu, Ian C. Davis, Elliott D. Crouser

**Affiliations:** 1 Division of Pulmonary, Critical Care and Sleep Medicine of the Department of Internal Medicine, The Ohio State University, Columbus, OH USA; 2 Center for Clinical and Translational Sciences, The Ohio State University, Columbus, OH USA; 3 Department of Biomedical Informatics, The Ohio State University, Columbus, OH USA; 4 Department of Veterinary Biosciences, The Ohio State University, Columbus, OH USA

**Keywords:** Lung, SARS CoV-2, COVID-19, Influenza, Acute respiratory failure, Hypoxemia, CDP-choline, Citicoline

## Abstract

**Background:**

The SARS CoV-2 pandemic has resulted in more than 1.1 million deaths in the USA alone. Therapeutic options for critically ill patients with COVID-19 are limited. Prior studies showed that post-infection treatment of influenza A virus-infected mice with the liponucleotide CDP-choline, which is an essential precursor for de novo phosphatidylcholine synthesis, improved gas exchange and reduced pulmonary inflammation without altering viral replication. In unpublished studies, we found that treatment of SARS CoV-2-infected K18-hACE2-transgenic mice with CDP-choline prevented development of hypoxemia. We hypothesize that administration of citicoline (the pharmaceutical form of CDP-choline) will be safe in hospitalized SARS CoV-2-infected patients with hypoxemic acute respiratory failure (HARF) and that we will obtain preliminary evidence of clinical benefit to support a larger Phase 3 trial using one or more citicoline doses.

**Methods:**

We will conduct a single-site, double-blinded, placebo-controlled, and randomized Phase 1/2 dose-ranging and safety study of Somazina® citicoline solution for injection in consented adults of any sex, gender, age, or ethnicity hospitalized for SARS CoV-2-associated HARF. The trial is named “SCARLET” (Supplemental Citicoline Administration to Reduce Lung injury Efficacy Trial). We hypothesize that SCARLET will show that i.v. citicoline is safe at one or more of three doses (0.5, 2.5, or 5 mg/kg, every 12 h for 5 days) in hospitalized SARS CoV-2-infected patients with HARF (20 per dose) and provide preliminary evidence that i.v. citicoline improves pulmonary outcomes in this population. The primary efficacy outcome will be the S_p_O_2_:F_i_O_2_ ratio on study day 3. Exploratory outcomes include Sequential Organ Failure Assessment (SOFA) scores, dead space ventilation index, and lung compliance. Citicoline effects on a panel of COVID-relevant lung and blood biomarkers will also be determined.

**Discussion:**

Citicoline has many characteristics that would be advantageous to any candidate COVID-19 therapeutic, including safety, low-cost, favorable chemical characteristics, and potentially pathogen-agnostic efficacy. Successful demonstration that citicoline is beneficial in severely ill patients with SARS CoV-2-induced HARF could transform management of severely ill COVID patients.

**Trial registration:**

The trial was registered at www.clinicaltrials.gov on 5/31/2023 (NCT05881135).

**Trial status:**

Currently enrolling.

**Supplementary Information:**

The online version contains supplementary material available at 10.1186/s13063-024-08155-0.

## Background

To date, there have been more than 100 million SARS CoV-2 coronavirus infections in the USA, resulting in more than 1.1 million deaths and counting. Effective vaccines are now available [1], but the virus continues to mutate and circulate within the population, and there is an ongoing risk of future severe pandemic waves.

Symptomatic SARS CoV-2 infection can progress to a clinical syndrome termed coronavirus disease of 2019 (COVID-19). Severe COVID-19 is characterized by acute respiratory failure and development of the acute respiratory distress syndrome (ARDS). COVID-19 may be complicated by coagulopathy, acute cardiac injury, renal injury, and other systemic manifestations [2–5]. Approximately 20% of all hospitalized patients [6] and up to 40% of those admitted to the intensive care unit (ICU) die [7]. Moreover, around 15% of survivors [8] develop “long COVID” [9], a disabling condition which is more likely after severe acute illness [10].

Treatment options for severely ill patients with COVID-19 are limited. Remdesivir, immunomodulators such as tocilizumab and baricitinib, and corticosteroids have been shown to be of some benefit and are currently recommended for use, but supportive ICU care remains central to managing these patients. Specifically, administration of high concentrations of supplemental O_2_ and mechanical ventilation are often required [11, 12]. Supportive care is resource-intensive, and interventions such as O_2_ therapy and mechanical ventilation can themselves be injurious to the lung [13, 14]. Additional therapeutics to manage patients with COVID-19 and reduce the need for ICU care are therefore urgently needed.

Approximately 50% of the epithelial cells lining the alveoli in the distal lung are small cuboidal ATII cells [15]. ATII cells regulate the depth of the alveolar lining fluid by alveolar fluid clearance [16]. They also synthesize, secrete, and recycle pulmonary surfactant proteins and lipids (including phospholipids), which help to maintain low alveolar surface tension [17], reducing dynamic alveolar collapse and preventing gas exchange impairment during ventilation. Surfactant phospholipids also have anti-inflammatory properties, and surfactant proteins play an important role in host defense against pathogens [18]. ATII cells are thus essential to normal lung function and host defense and play a central role in ARDS pathogenesis.

ATII cell dysfunction and death in ARDS results in reduced surfactant production and function, leading to poor lung compliance, predisposition to ventilator-induced lung injury (VILI), impaired pathogen clearance, and enhanced inflammation [19–24]. ARDS also results in impaired alveolar fluid clearance [25], which is associated with a poor prognosis [11, 26]. Importantly, human ATII cells express the receptor for CoV-2 (ACE2) [27] and the protease cofactor TMPRSS2 [28] and SARS CoV-2 antigens have been detected in ATII cells from COVID-19 autopsy samples [29–33]. SARS CoV-2 has also been shown to replicate in ATII cells in vitro [34–36]. In addition to inducing release of inflammatory mediators [37], SARS CoV-2 infection of ATII cells may result in disruption of surfactant synthesis and alveolar fluid clearance, both of which may contribute significantly to development of COVID-19 [38, 39].

The phospholipid dipalmitoyl-phosphatidylcholine (16:0/16:0) is the largest component of pulmonary surfactant [40]. Dipalmitoyl-phosphatidylcholine is synthesized by the Kennedy pathway [41]. Synthesis of the liponucleotide cytidine 5’-diphospho (CDP)-choline from choline phosphate and CTP by the enzyme CTP-phosphocholine cytidylyltransferase-α (CCT-α) is rate limiting for this pathway. We showed that murine ATII cell CDP-choline synthesis is rapidly and completely inhibited by IAV infection in vivo [42]. Although we have not yet shown that SARS CoV-2 also inhibits ATII cell CDP-choline synthesis, multiple groups have reported that plasma or serum from COVID-19 patients contains reduced phosphatidylcholine [43–48], suggesting that SARS CoV-2 disrupts this pathway. Moreover, some of the beneficial effects of dexamethasone in COVID-19 patients could be because it stimulates CCT-α activity [49–56], as does CDP-choline [57].

We hypothesize that impaired ATII cell CDP-choline synthesis is a common feature of viral ARDS, which contributes significantly to its pathogenesis. Hence, we propose that the de novo phospholipid synthesis pathway is a promising target for host-directed therapy for viral ARDS. In support of this, we have shown that CDP-choline strongly attenuates IAV-induced ARDS and pulmonary inflammation [58] and prevents hypoxemia in SARS CoV-2-infected male K18-hACE2-Tg mice (unpublished data). Hence, we propose that this compound will be effective in COVID-19 patients. The pharmaceutical form of CDP-choline is citicoline.

SCARLET has two goals. Firstly, we will show that i.v. citicoline administration is safe over a range of doses in hospitalized SARS CoV-2-infected patients with hypoxemic acute respiratory failure (HARF). Secondly, we will obtain preliminary evidence that i.v. citicoline improves outcomes in this patient population relative to current standard of care and identify the recommended dose and appropriate clinical endpoints for larger Phase 3 efficacy trials. We will use a placebo control to represent current standard of care. We expect to find that citicoline is safe at all tested doses in COVID-19 patients. We also expect to find that citicoline has clinical benefit at one or more doses.

## Methods

### Participants, interventions, and outcomes

#### Study design and setting

**Fig. 1:**
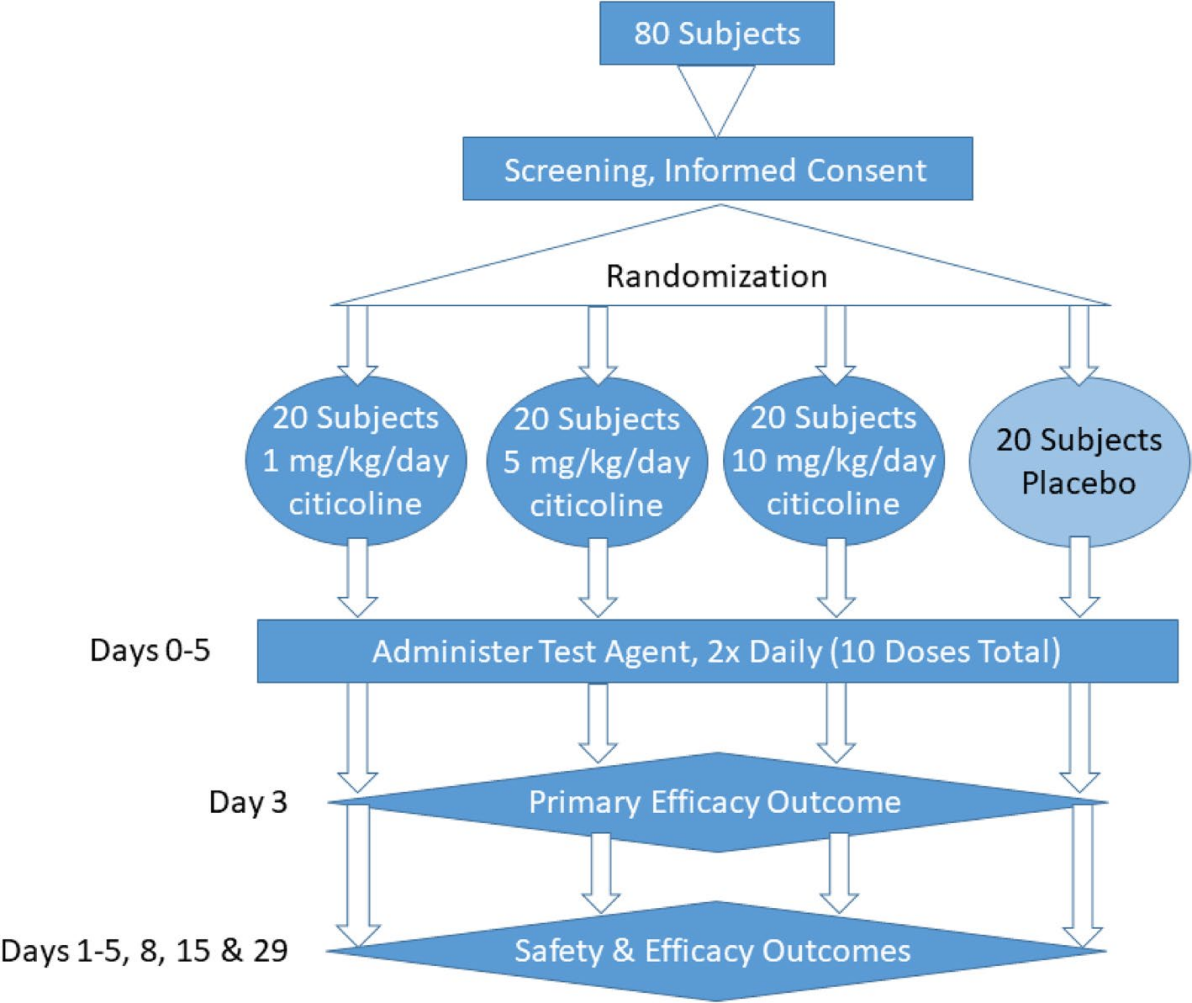
Trial design. SCARLET is a single-center, double‐blinded, placebo‐controlled, and randomized Phase 1/2 trial of i.v. citicoline in adult patients of any sex, gender, age, or ethnicity who are hospitalized with HARF following SARS CoV‐2 infection. The trial will enroll 20 patients per dose for 3 citicoline doses (1, 5, and 10 mg/kg/day, split into 2 doses 12 h apart) along with 20 placebo (sterile saline)‐treated controls

SCARLET is a single-center, double‐blinded, placebo‐controlled, and randomized Phase 1/2 trial of i.v. citicoline in adult patients of any sex, gender, age, or ethnicity who are hospitalized with HARF following SARS CoV‐2 infection (Table 1). Patients will be enrolled at The Ohio State University (OSU) Wexner Medical Center (OSU WMC) and OSU WMC Hospital East (OSU East), both of which are in Columbus, Ohio, USA. The goals are to confirm citicoline safety over a range of doses and demonstrate potential for efficacy in this population for at least one citicoline dose. The trial will enroll 20 patients per dose for 3 citicoline doses (0.5, 2.5, and 5 mg/kg every 12 h for 5 days) along with 20 placebo‐treated controls (See Fig. 1).

**Table 1:** WHO trial registration dataset

**Primary Registry and Trial Identifying Number**	Clinicaltrials.gov: NCT05881135
Date of Registration in Primary Registry	5/31/2023
Secondary Identifying Numbers	Federal Award Identification Number: U01AI677784 FDA IND: 162,806 OSU IRB protocol number: 2022H0451
Source of Monetary Support	NIAID Co-operative agreement
Primary Sponsor	Elliott Dr. Crouser, MD
Secondary Sponsor(s)	None
Contact for Public Queries	Elliott Dr. Crouser, MD Professor, Dept. of Internal Medicine, The Ohio State University elliott.crouser@osumc.edu 614–346-6690
Contact for Scientific Queries	Principal Investigators: Elliott Dr. Crouser, MD Professor, Dept. of Internal Medicine, The Ohio State University elliott.crouser@osumc.edu 614–346-6690 2050 Kenny Road Suite 2600 Columbus, OH 43221 USA Ian C. Davis, DVM, PhD, ATSF Professor, Dept. of Veterinary Biosciences, The Ohio State University davis.2448@osu.edu 614–483-5615 307 Goss Labs 1925 Coffey Road Columbus, OH 43210 USA
Public Title	Citicoline for hospitalized COVID patients
Scientific Title	SCARLET (Supplemental Citicoline Administration to Reduce Lung injury Efficacy Trial)
Countries of Recruitment	United States of America
Health Condition Studied	COVID-19
Intervention	Investigative agent: 0.5, 2.5, or 5 mg/kg) of Somazina® citicoline (CDP-choline) solution for injection (Ferrer Internacional, S.A., Barcelona, Spain) diluted in sterile USP-grade normal saline to 10 ml final volume, administered i.v. every 12 h for 5 days Placebo: 10 ml sterile USP-grade normal saline, administered i.v. every 12 h for 5 days
Key Inclusion and Exclusion Criteria	Inclusion Criteria: (1) Hypoxemic acute respiratory failure plus evidence of active SARS CoV-2 infection (positive for SARS CoV-2 using antigen or PCR test within the 10 days prior to randomization) (2) C-reactive protein > 32 mg/dl Exclusion Criteria: (1) Prisoners (2) Women who may be pregnant, are pregnant, or who are breast feeding (3) Subjects who are unable or unwilling to give written informed consent or to comply with study protocol and who have no legal authorized representative (LAR) available to give consent on their behalf (4) Individuals with a known allergy to citicoline (5) Subjects that are taking medications that contain L‐Dopa, centrophenoxine, or meclofenoxate (6) Individuals with hypertonia of the parasympathetic nervous system (7) Subjects who, in the clinicians estimation, will be unlikely to survive the protocol duration due to imminent and unavoidable risk of death (8) Individuals being treated with extracorporeal membrane oxygenation (ECMO) (9) Individuals with multiple organ failure
Study Type	Single-site, double-blinded, placebo-controlled, and randomized Phase 1/2 dose-ranging and safety study
Date of First Enrollment	7/14/2023
Sample Size	Planned: 80 Enrolled: 15
Recruitment Status	Recruiting
Primary Outcome	A statistically significant difference in lowest recorded S_p_O_2_:F_i_O_2_ ratio on study day 3, at *P* < 0.05
Key Secondary Outcomes	Statistically significant differences (*P* < 0.05) in: (1) Lowest recorded S_p_O_2_:F_i_O_2_ value on study days 1–2 and 4–8, or until extubation (whichever comes first) (2) Lowest Sequential Organ Failure Assessment (SOFA) score on study days 1, 3, 5, and 8 (3) Highest COVID Ordinal Outcomes Scale score on study days 1, 3, 8, 15, and 29 Oxygen-free days through day 28 Ventilator-free days through day 28 ICU-free days through day 28 Hospital-free days through day 28
Ethics Review	Approved by The Ohio State University Biomedical Sciences Institutional Review Board on 3/28/23 300 Research Administration building 1960 Kenny Road Columbus, OH 43210–1063 irbinfo@osu.edu
IPD sharing statement	Plan to share IDP: Yes All data will be deposited to the Dataverse that is supported by Harvard University under waivers providing for public availability of data repository starting 12 months after the trial begins and will be deposited every six months thereafter. This repository provides metadata, persistent unique identifiers, and long-term access for at least 10 years

#### Eligibility criteria

Adult patients (≥ 18 years) of both sexes and any race or ethnicity will be eligible for enrollment. Research personnel (physicians and research coordinators) will screen patients daily for eligibility daily under partial HIPAA waiver using the electronic medical record (EMR; Epic, Madison, WI, USA) to facilitate identification of SARS CoV-2-positive patients who may meet other eligibility criteria for the trial. Enrollment is feasible any time during the hospital stay; however, if hospital discharge is anticipated within 24 h the patient will not be enrolled. Patients previously screened and not meeting inclusion criteria or with exclusion criteria may be rescreened daily until eligibility expires (> 10 days post admission).

Initial screening will assess for evidence of HARF plus evidence of active SARS CoV-2 infection. Only patients positive for SARS CoV-2 using antigen or PCR test within the 10 days prior to randomization will be eligible. Patients must also have a C-reactive protein > 32 mg/dl. Assurance of adequate peripheral or central venous access is a requirement. Female subjects of childbearing potential must have a negative pregnancy test upon study entry.

Patients meeting inclusion criteria will be screened for exclusion criteria. Individuals who meet any of these criteria are not eligible for enrollment as study participants: (1) Prisoners; (2) Women who may be pregnant, are pregnant, or who are breast feeding; (3) Subjects who are unable or unwilling to give written informed consent or to comply with study protocol and who have no legal authorized representative (LAR) available to give consent on their behalf; (4) Individuals with a known allergy to citicoline; (5) Subjects that are taking medications that contain L‐Dopa, centrophenoxine, or meclofenoxate; (6) Individuals with hypertonia of the parasympathetic nervous system; (7) Subjects who, in the clinicians estimation, will be unlikely to survive the protocol duration due to imminent and unavoidable risk of death; (8) Individuals being treated with extracorporeal membrane oxygenation (ECMO); (9) Individuals with multiple organ failure; and/or (10) Subjects with past or current medical problems or findings from physical examination or laboratory testing that are not listed above, which, in the opinion of the principal investigator (PI; E.D.C.), may pose additional risks from participation in the study or that may impact the quality or interpretation of the data obtained from the study.

Critically ill children with SARS CoV-2 infection generally present with severe multisystem inflammatory syndrome (MIS-C) requiring vasoactive drugs and immunomodulators and are less likely to need ventilatory support than adults with severe COVID-19, who primarily develop progressive HARF which often leads to ARDS [59–62]. Hence, children will not be enrolled.

Following screening, eligible patients will be discussed with the patient’s primary care team. The ability to provide consent, directly or by a surrogate decision maker, will then be determined among patients who are deemed eligible. The patient or their LAR must be able to understand and provide informed consent. For subjects who are eligible for enrollment but are not recruited based on the clinician’s opinion that participation may not be in their best interests, the stated reason and name of the physician making the decision not to enroll will be recorded.

After approval from their care team, one of the study physicians or their official designee (a research coordinator) will approach the patient or their LAR if the patient is deemed unable to provide consent. The research study will be explained in lay terms in accordance with OSU standard operating procedures. Disclosures of potential conflicts of financial interest for the Institution and for I.C.D. (who will not be involved in patient interactions or treatment decisions) will be included in the final consent form. Once informed consent has been obtained, subjects will be enrolled into the study and randomized using random block permutations. Randomization will be done by OSU WMC Investigational Drug Services (IDS) and all clinical and research personnel will be blinded to allocation. Randomization must occur within 24 h from time of consent.

A participant will be deemed to have completed the study following administration of their final dose of test agent (10 doses over 5 days), unless discharged earlier. Study participation may be prematurely terminated for the following reasons: (1) The participant elects to withdraw consent from all future study activities, including follow‐up; (2) The participant dies; (3) The PI no longer believes participation is in the best interest of the participant; or (4) The PI believes that a serious adverse event (SAE) in the patient may be attributable to the investigational agent.

Participants who withdraw or are withdrawn will not be replaced if they have received at least one dose of the investigational agent. No follow-up will occur for participants who withdraw or are withdrawn from the trial.

#### Informed consent

The consent process will provide information about the study to a prospective participant and will allow adequate time for review and discussion prior to his/her/their decision. The PI will review the consent with the patient or their LAR and answer questions. The prospective participant will be told that being in the trial is voluntary and that they may withdraw from the study at any time, for any reason. All participants (or their LAR) will read, sign, and date a consent form before undergoing any study procedures. Consent materials will be presented in participants’ primary language. A copy of the signed consent form will be given to the participant.

The consent process will be ongoing. The consent form will be revised when important new safety information is available, the protocol is amended, and/or new information becomes available that may affect participation in the study.

### Interventions

#### Intervention description

Patients will be randomized 1:1:1:1 to receive one of 3 doses (0.5, 2.5, or 5 mg/kg) of Somazina® citicoline solution for injection (Ferrer Internacional, S.A., Barcelona, Spain) diluted in sterile normal saline or placebo (sterile normal saline). Somazina® is approved by the European Medicines Agency for use with a prescription in Europe. Patients will be treated with study agent every 12 h for up to 5 days.

Citicoline will be provided in sterile, single-use, Type I neutral glass ampoules containing 1000 mg active pharmaceutical ingredient (citicoline) dissolved in 4 ml water (final concentration 250 mg/ml), with HCl added to normalize pH; no other excipients are present. Study ampoules will be shipped from Ferrer directly to the IDS at OSU WMC and stored at room temperature in the dark. The immediate package of this investigational new drug will be labeled with the statement “Caution: New Drug – Limited by Federal (or United States) law to investigational use.”

Study drugs will be prepared on the day of use by a research pharmacist in the IDS at OSU WMC. Citicoline solution will be diluted in USP-compliant sterile normal saline to a final volume of 20 ml containing the appropriate total daily dose for each patient (1, 5, or 10 mg/kg/day, based on their ID number). Doses will be rounded to the nearest 0.1 ml per standard rounding rules. Two 10-ml aliquots will then be prepared in sterile disposable syringes labeled with the patient ID number for administration 12 h apart and then kept refrigerated. Unused undiluted or diluted citicoline will be discarded by the IDS pharmacist and documented as such. Placebo controls will receive 10 ml USP-compliant sterile normal saline every 12 h for 5 days.

Each 10 ml dose of test agent will be delivered by nursing staff as a slow i.v. bolus over 3–5 min and immediately documented in the EMR. EMRs will be reviewed daily by the clinical trial coordinator to ensure test agent administration has occurred and been documented.

#### Criteria for discontinuing or modifying allocated interventions

No modifications to the test agent dose or frequency will be permitted during the study period. Study therapy may be prematurely discontinued for any participant for any of the following reasons: (1) SAEs occur that, in the view of the attending clinician, study investigator, or PI, may be attributable to citicoline; (2) The PI believes that the study treatment is no longer in the best interest of the participant; or (3) The Data Safety Monitoring Board (DSMB) will make a determination of futility or potential for harm to the patient(s).

#### Strategies to improve adherence to interventions

All patients enrolled in the study will be hospitalized and will therefore receive monitoring by their physicians, nurses, respiratory therapists, and ancillary staff as a part of routine clinical care. A drug‐dispensing log will be kept current for each participant. This log will contain the identification of each participant and the date and quantity of drug dispensed. Under Title 21 of the Code of Federal Regulations (21CFR §312.62), the PI will maintain adequate records of the disposition of the investigational agent, including the date and quantity of the drug received, to whom the drug was dispensed (participant‐by‐participant accounting), and a detailed accounting of any drug accidentally or deliberately destroyed. Records for receipt, storage, use, and disposition will be maintained by the IDS and will be available for inspection.

#### Relevant concomitant care permitted or prohibited during the trial

Enrolled participants will not receive open-label citicoline during the 5-day intervention period. All other treatment decisions will be made by treating clinicians without reference to the protocol. Treatment of the underlying SARS CoV-2 viral infection is left to the discretion of the treating team. Depending on disease severity, and as based on current recommendations from the NIH, treatment may include but is not limited to antiviral therapy with remdesivir and administration of anti-inflammatory agents such as dexamethasone or tocilizumab. Patients deemed high risk for venothromboembolic disease may be treated with either prophylactic or therapeutic anticoagulation as appropriate. Other common medications such as vasopressors, sedative agents, antimicrobials, and neuromuscular blockade will be used in accordance with local standard of care in accordance with best practices.

Serious adverse reactions to citicoline have not been reported in the medical literature and are considered unlikely. Nevertheless, between randomization and day 5, study personnel will review the EMR daily for potential medication interactions with citicoline. There are no rescue medications for citicoline per se; however, reported side effects include headache, nausea, and low blood pressure. Rescue therapies for these side effects include (1) Analgesics for headache (acetaminophen, ibuprofen, rarely narcotics); (2) Antiemetics (ondansetron, metoclopramide); and (3) Fluid bolus for low blood pressure. Should a patient develop evidence of a severe reaction, resuscitation and treatment will be at the discretion of the treating physicians. Specifically, study drug will be discontinued at the time of suspicion of an adverse reaction. If anaphylaxis is suspected, the patient will receive epinephrine, anti‐histamines, and corticosteroids as appropriate. Resuscitation for hypotension will occur with fluids or vasopressor agents.

Rescue medications themselves can cause adverse reactions. Dexamethasone often causes transient hyperglycemia and may increase susceptibility to secondary infection of the lungs. Remdesivir may predispose to reversible transaminase elevation and case reports of bradycardia have been seen in post-market analysis. Mild transaminase elevation has been seen after administration of tocilizumab, so liver function monitoring is recommended. Immune suppression is expected following corticosteroid and tocilizumab dosing, and patients will be monitored for secondary bacterial infections.

#### On-study monitoring

Peripheral arterial O_2_ saturation (S_p_O_2_) will be monitored continuously and recorded every 2 min. Arterial blood gases will be measured on study days 1, 3, 5, and 8, or as clinically necessary.

Venous blood will be drawn at Time 0 and then once daily on study days 1–5 and 8 and processed by a research coordinator. At each study timepoint, a sufficient volume of venous blood will be drawn into blood container tubes containing appropriate anticoagulants to complete all the following assays: (1) CBC/differential count; (2) Hematology panel; (3) Clinical chemistry panels for renal, cardiac, and hepatic function; (4) Prothrombin Time and Partial Thromboplastin Time; (5) D-dimer, ferritin, fibrinogen; and (6) Thromboelastography. These assays will be performed by the clinical lab at the hospital in which that subject is located. All OSU hospital clinical laboratories are Clinical Laboratory Improvement Amendment-approved by the Dept. of Health and Human Services and are accredited by the College of American Pathologists’ Laboratory Accreditation Program. All clinical laboratory testing for study participants will comply with 42 CFR part 493.2 and 493.3(b)(2).

An additional 10 ml heparinized venous blood will be collected from each subject at each study timepoint for research purposes. Venous blood collected for research use from patients at both OSU East and OSU Main will be transferred to Dr. Crouser’s laboratory in the Davis Heart and Lung Research Institute (part of OSU WMC and an approved research space). Plasma will be divided into 1-ml aliquots and stored in a locked − 80 °C freezer for batch analysis of biomarkers. Leukocytes will be resuspended in freezing media, aliquoted into cryovials, and stored at − 80 °C for downstream research studies.

Chest X-rays will be obtained on admission and subsequently when clinically indicated: for example, to confirm suspected secondary infections, etc. Chest X-ray is also routine after certain procedures (placing of central lines, intubation, thoracentesis, etc.). Subjective scoring by quadrants will be performed to determine whether this may be a useful secondary outcome measure for Phase 3 trials.

#### Biomarker analysis

Effects of citicoline on a variety of potential plasma biomarkers of ARDS and/or COVID-19 severity will be determined. Plasma IL-6, IL-8, IL-10, IL-18, and other cytokines and chemokines in plasma will be measured by Bio-Plex (48-Plex Human Cytokine Screening Panel). IFN-α, IFN-λ, Angiopoietin 2, C-reactive protein, lactate dehydrogenase, RAGE, and SP-D will be assayed using commercial ELISA kits. Plasma viremia, which has also been linked to prognosis [63, 64], will be quantified by qRT-PCR for SARS CoV-2 N gene using the CDC protocol. Batched assays will be performed in accordance with Good Laboratory Practice principles using validated commercial reagents. Residual samples will be retained in the − 80 °C freezer until conclusion of the study and analysis and then destroyed according to OSU protocol.

#### Outcomes

The primary goals of SCARLET are to establish the safety of citicoline in hospitalized SARS CoV-2-infected patients with HARF, to generate preliminary evidence of efficacy, and to identify an optimal citicoline dose for Phase 3 efficacy trials. There are therefore both safety- and efficacy-related primary outcome measures.

A particular dose of citicoline will be considered safe if the number of SAEs attributable to that citicoline dose (in the view of treating clinicians) remains below the threshold indicative of excessive toxicity once all 20 subjects have been treated (see Table 2).

**Table 2:** Number of patients with SAEs required to claim excessive toxicity

No. of patients	1–2	3–4	5–7	8–10	11–14	15–17	18–20
No. with SAEs	–	2	3	4	5	6	7

The primary clinical outcome is a statistically significant difference in lowest recorded S_p_O_2_:F_i_O_2_ (S:F) ratio on study day 3, at *P* < 0.05. The lowest S_p_O_2_ for the day will be identified and used to generate the S:F ratio (based on the F_i_O_2_ at time of lowest S_p_O_2_ value).

Secondary/exploratory outcomes will be evaluated to identify statistically significant differences (*P* < 0.05) in (1) Lowest recorded S:F value on study days 1–2 and 4–8, or until extubation (whichever comes first); (2) Lowest Sequential Organ Failure Assessment (SOFA) score on study days 1, 3, 5, and 8; and (3) Highest COVID Ordinal Outcomes Scale score on study days 1, 3, 8, 15, and 29. On study day 29, we will also assess (1) Oxygen-free days through day 28; (2) Ventilator-free days through day 28; (3) ICU-free days through day 28; and (4) Hospital-free days through day 28.

#### Participant timeline

The timing of study procedures is based on the time at which consent was obtained, which is defined as “Time 0”. Chest X-ray will be performed and baseline Sequential Organ Failure Assessment (SOFA) and COVID Ordinal Outcomes Scale (HRS) scores will be calculated at Time 0. Study test agents will be administered by study personnel while the patient is hospitalized, with the first dose being administered within 4 h of Time 0. If the patient is discharged prior to completion of the study medication, no further study medication will be administered.

**Fig. 2:**
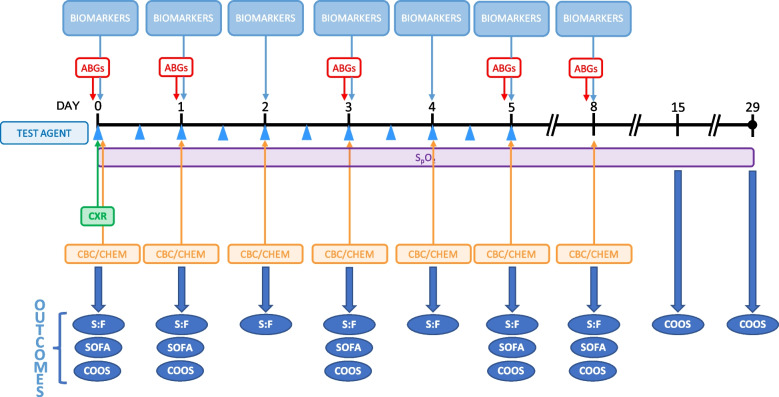
Individual patient schedule. Patients will receive test agent (citicoline or saline placebo) every 12 h for 5 days. Patients will be monitored out to day 29. ABGs—arterial blood gases; CBC/CHEM—complete blood count and chemistry panel; COOS—COVID Ordinal Outcomes Scale; CXR—chest X-ray; S:F—S_a_O_2_:F_i_O_2_ ratio; SOFA—Sequential Organ Failure Assessment

Individual patient schedules are shown below and in Fig. 2.

Day 0–1:

1. Informed consent will be obtained.
2. Order for study drug will be placed by the investigator.
3. Initial clinical data will be obtained by research staff using the EMR and recorded in the REDCap study database. Missing laboratory data will be ordered by the investigator.
4. Blood will be drawn from all patients prior to the first treatment dose for laboratory studies.
5. Study drug will be administered i.v. by the bedside nurse operating within their usual scope of practice. The timing of study drug administration may be adjusted up to 4 h on subsequent dosing to achieve standard dosing times.
6. Clinical information, including other drugs administered, will be recorded by the research team into the REDCap study database.

Days 1–5:

1. The patient will be assessed daily by study team for evidence of adverse drug reactions.
2. Dosing with the test agent will continue every 12 h until each subject has received 10 doses total.
3. Laboratory samples will be collected according to the study timeline.
4. Clinical information, including other drugs administered, will be recorded by the research team into the REDCap study database.

Days 6–8:

1. The patient will be assessed daily by study team for evidence of adverse drug reactions or clinical change.
2. Laboratory samples will be collected according to the study timeline.

Clinical information, including other drugs administered, will be recorded by the research team into the REDCap study database.

Days 9–29:

1. Patient will be assessed daily by study team for evidence of adverse drug reactions or clinical change.
2. Patients discharged home prior to day 29 will be called on day 29 to verify vital status and discharge location.
3. Patients who withdrew from the trial at any point prior to the cessation of test agent administration

#### Participant stopping rules and withdrawal criteria

Participants may be prematurely terminated from the study for the following reasons: (1) The participant elects to withdraw consent from all future study activities, including follow‐up; (2) The participant dies; (3) The Investigator no longer believes participation is in the best interest of the participant; or (4) The Investigator believes that an SAE in the patient may be attributable to the test agent.

Participants who withdraw or are withdrawn will not be replaced if they have received at least one dose of the investigational agent.

Patients wishing to withdrawal early from the study (i.e., before all 10 doses were administered) will be asked if they are agreeable to follow-up phone call on day #29. If approval is granted, they will be called on day 29 to verify vital status and discharge location. Patients opting complete withdrawal from the study will not be contacted further.

#### Protocol modifications

Any modifications to the approved clinical protocol will be developed in consultation with the DSMB and will be implemented only after approved by the OSU IRB and FDA. Once the protocol change is approved by the IRB, the DSMB and NIAID will be notified.

The PI will then schedule an educational session to review changes in the protocol with the participation of each team member recorded.

1. Sample size. Sample size calculations are based on differences in % carotid S_p_O_2_ between saline and CDP‐choline treatment groups in SARS CoV‐2-infected mice (unpublished data). Effect sizes post treatment were 1.32 in IAV and 1.4 in SARS CoV‐2 infected mice. With 24 subjects in the 2.5 and 5 mg/kg/dose treatment groups and 24 controls, and allowing 5% missing data at day 3 (80 participants total), there is 80% power (*α* = 0.05) to detect an effect size of 0.9 in S:F ratio between any individual arm and control (0.39 effect size overall), 32% below that in our preliminary data.

### Safety events

#### Definitions

An adverse event (AE) will be defined as any untoward or unfavorable medical occurrence associated with the subject’s participation in SCARLET, whether or not considered related to the subject’s participation in the trial (modified from the definition of AEs in the 1996 International Conference on Harmonization E‐6 Guidelines for Good Clinical Practice). For this study, an AE will include any untoward or unfavorable medical occurrence associated with (1) The Study therapy regimen (any AE occurring after initial dosing of study drug until hospital discharge or study day 29 whichever is longer); or (2) Study-mandated procedures (any AE associated within 12 h of study-mandated phlebotomy).

Severity of AEs experienced by the study subjects will be graded according to the criteria set forth in the National Cancer Institute’s Common Terminology Criteria for Adverse Events (CTCAE) version 5.0. This manual provides a common language to describe levels of severity, to analyze and interpret data, and to articulate the clinical significance of all AEs. AEs will be graded Mild (Grade 1), Moderate (Grade 2), Severe but not immediately life threatening (Grade 3), or Severe with life‐threatening consequences requiring urgent intervention (Grade 4). An AE-related death is considered Grade 5. For grading an abnormal value or result of a clinical or laboratory evaluation, a treatment‐emergent AE is defined as an increase in grade from baseline or from the last post‐baseline value that does not meet grading criteria. Changes in grade from screening to baseline will also be recorded as AEs but are not treatment‐emergent. An abnormal result would also be considered an AE if changes in therapy or monitoring are implemented as a result of the event/result.

A suspected adverse reaction (SAR) will be defined as any AE for which there is a reasonable possibility that the investigational drug caused the AE. For the purposes of safety reporting, “reasonable possibility” means there is evidence to suggest a causal relationship between the drug and the AE. A suspected adverse reaction implies a lesser degree of certainty about causality than adverse reaction, which means any AE caused by a drug (21 CFR 312.32(a)).

An AE or serious adverse reaction (SAR) will be considered “unexpected” if it is not listed in the package insert. An AE or SAR is considered “serious” if, in the view of either the PI or DMSB, it results in any of the following outcomes (21 CFR 312.32(a)): (1) Death; (2) A life‐threatening event (an AE or SAR is considered “life‐threatening” if, in the view of the Investigator, the Medical Monitor, or the DMSB, its occurrence places the subject at immediate risk of death; it does not include an AE or SAR that, had it occurred in a more severe form, might have caused death); (3) Prolongation of existing hospitalization; (4) Persistent or significant incapacity or substantial disruption of the ability to conduct normal life functions; (5) A congenital anomaly or birth defect; or (6) Any other important medical events that may not result in death, be life threatening, or require hospitalization but may be considered serious when, based upon appropriate medical judgment, they may jeopardize the subject and may require medical or surgical intervention to prevent one of the outcomes listed above.

#### Recording of AEs

AEs will be collected from the time of first dose of study drug, until a subject completes study participation (day 29) or until 30 days after they prematurely withdraw (without withdrawing consent) or is withdrawn from the study. AEs (including SAEs) may be discovered through any of these methods: (1) Observing the subject; (2) Interviewing the subject; or (3) Receiving an unsolicited complaint from the subject. In addition, an abnormal value or result from a clinical or laboratory evaluation can also indicate an AE.

Throughout the study, the PI and his team will record Grade 2 or higher AEs on the appropriate AE/SAE or electronic case report form (eCRF) regardless of the relationship to study therapy regimen or study procedure. Once recorded, an AE will be followed until it resolves with or without sequelae, or until the end of study participation, or until 30 days after the subject prematurely withdraws (without withdrawing consent)/or is withdrawn from the study, whichever occurs first.

The relationship, or attribution, of an AE to the study therapy regimen or study procedure(s) will initially be determined by the PI and recorded on the appropriate AE (AE/SAE or eCRF). Final determination of attribution for safety reporting will be determined by the Medical Monitor, DSMB, PI, and NIAID*.*

#### Reporting of AEs and SAEs

The PI shall report any suspected adverse reaction that is both serious and unexpected, as described below. The PI shall report an AE as a suspected adverse reaction only if there is evidence to suggest a causal relationship between the study drug and the AE, such as (1) a single occurrence of an event that is uncommon and known to be strongly associated with drug exposure (e.g., angioedema, hepatic injury, or Stevens‐Johnson Syndrome); (2) one or more occurrences of an event that are not commonly associated with drug exposure, but are otherwise uncommon in the population exposed to the drug (e.g., tendon rupture); (3) an aggregate analysis of specific events observed in a clinical trial (such as known consequences of the underlying disease or condition under investigation or other events that commonly occur in the study population independent of drug therapy) that indicates those events occur more frequently in the drug treatment group than in a concurrent or historical control group.

Worsening respiratory function, including but not limited to need for increased oxygen or mechanical ventilation, occurs commonly in this study population and will not be categorized as an SAE if deemed by treating team to be consistent with progression of known COVID-19, unless there is evidence to suggest a causal relationship to citicoline. These events will be captured in the study database but will not be reported as expedited Safety Reports:

Timely reporting of AEs is required by 21 CFR and ICH E6 guidelines. The PI will report all SAEs regardless of relationship or expectedness within 24 h of discovering the event. Other AEs, including expedited reports, will be reported in a timely fashion to the OSU IRB in accordance with applicable regulations and guidelines. The DSMB Annual Study Report to health authorities will include all AEs classified as serious, expected, or suspected. All Safety Reports to the FDA shall be distributed by the DSMB or designee for submission to the OSU IRB.

For SAEs, all requested information on the AE/SAE eCRF will be provided. However, unavailable details of the event will not delay submission of the known information. As additional details become available, the AE/SAE eCRF will be updated and submitted.

The sponsor shall notify the FDA and all participating investigators of Expedited Safety Reports within 15 calendar days; unexpected fatal or immediately life‐threatening suspected adverse reaction(s) shall be reported as soon as possible or within 7 calendar days to appropriate health authorities. The sponsor shall also report any findings from other epidemiological studies, analyses of AEs within the current study or pooled analysis across clinical studies or animal or in vitro testing (e.g., mutagenicity, teratogenicity, carcinogenicity) that suggest a significant risk in humans exposed to the drug that would result in a safety‐related change in the protocol, informed consent, or other aspects of the overall conduct of the study.

#### Interim safety analysis

After enrollment of 8 subjects per group, an interim safety analysis will be performed based on a continuous safety monitoring rule to guide accrual suspension decisions based on unacceptable toxicity and SAEs. The number of patients with an SAE that would warrant temporary suspension of accrual in a given group corresponds to a high posterior probability that the true SAE probability is greater than an acceptable level (i.e., Pr(${p}_{i}$ > 0.25 | data) > 0.85), where the posterior probability is determined from a Beta-Binomial distribution with Beta (1, 1) as the prior on ${p}_{i}$. This is illustrated in Table 2.

For example, if 9 patients have been treated and 4 or more patients have an SAE, then accrual will be suspended while data are reviewed more closely and shared with the DSMB so that consensus regarding temporary suspension of accrual, protocol modifications, or decision to close the study early is made. Administration of the blinded study drug may be stopped temporarily or permanently for SAEs attributable to citicoline (in the view of the attending clinician) and in the event of clinical deterioration. In the latter case, the primary treating team is empowered to decide whether to stop the study drug and unblind group assignment. SAEs and unblinding will be recorded and reported to the DSMB at DSMB reviews and interim analyses.

#### Care provisions for patients experiencing harm

In the unlikely event a participating subject experiences an injury from participating in this study, the cost for any required treatment will be billed to that patient or their medical or hospital insurance. The Ohio State University has no funds set aside for the payment of health care expenses for this study.

### Randomization and blinding

Eligible participants who have provided informed consent will be randomized 1:1:1:1 to 0.5, 2.5, or 5 mg/kg/dose citicoline or placebo. Randomization will be completed in permuted blocks of variable size. Each subject will receive a computer-generated randomization ID number, which will be provided to the research pharmacy, where the appropriate test agent will be prepared. Test agent will be labeled with the ID number and date only.

Patients, treating clinicians, trial personnel, and outcome assessors will be blinded to group assignment until after the database is locked and blinded analysis is completed.

### Data management

#### Data collection plan

Data will be collected and managed using REDCap (Research Electronic Data Capture), which is a secure, web‐based electronic system for data entry, integration, processing, and reporting^(144, 145)^. In addition to generating and tracking on‐line case report forms, REDCap allows data validation (through checks on variable formatting and range and logic checks), query generation and tracking, and reporting. REDCap is easy to navigate and has been historically vetted for security and HIPAA compliance. REDCap also has functionality to store source documents, enabling staff and PI access to key study documents and improving operational workflows. Data access will be defined and tightly controlled according to each person’s role (blinded, unblinded). Advanced data filtering functions will ensure that staff have access to appropriate data while reducing the likelihood of unblinding. Data and data labels can be downloaded selectively (for interim progress reports) or in their entirety (at end of study) directly from REDCap in SAS or Excel format.

Data will be collected prospectively by study staff using the EMR. Following verification, data will be locked. Data access will be defined and tightly controlled according to each person’s role (blinded, unblinded).

#### Quality assurance and quality control

The clinical research manager will perform internal quality management of study conduct, data and biological specimen collection, documentation, and completion. Quality control procedures will be implemented beginning with the data entry system and data quality control checks that will be run on the database will be generated. Any missing data or data anomalies will be communicated to the PI for clarification/resolution.

Following written Standard Operating Procedures, study monitors will verify that the clinical trial is conducted, data are generated, and biological specimens are collected, documented (recorded), and reported in compliance with the protocol, International Conference on Harmonisation Good Clinical Practice, and applicable regulatory requirements (e.g., Good Laboratory Practices and Good Manufacturing Practices).

#### Access to source data

Documentation of source data is necessary for the reconstruction, evaluation and validation of clinical findings, observations, and other activities during a clinical trial. The original documentation where subject information, visits consultations, examinations, and other information are recorded are all considered source documents and source data. The PI and site staff will make all source documents, data, and reports available for inspection to the NIAID, as well as to relevant local and regulatory authorities for the purpose of monitoring and auditing. All representatives of these entities are bound to maintain the strict confidentiality of medical and research information that may be linked to identified individuals.

### Statistical analysis

All analysis will be based on intention to treat guidelines and reporting will follow CONSORT guidelines [65]. Data will be analyzed using STAT software (SAS; Cary, NC). The primary clinical outcome (S:F ratio on day 3) will be analyzed using either analysis of variance or a non-parametric Kruskal–Wallis test if analysis of variance assumptions (normality, homoscedasticity) are violated. Follow-up contrasts comparing each dose to control will be performed at *α* = 0.05/3 if the global test of differences between groups is rejected. If subject dropout due to mortality or discharge is present prior to day 3, the survivor average causal effect will employ a potential outcomes approach to account for potential survival bias and estimate the average causal effect of the intervention on S:F ratio among participants who would survive regardless of their intervention status, e.g., the always-survivors [66, 67]. The recommended citicoline dose for Phase 3 trials will be the dose achieving the greatest statistically significant improvement in day 3 S:F ratios, with consideration of key secondary endpoints (SOFA scores, etc.). Standardized mean differences will assess balance on baseline covariates across treatment arms. Competing risks analysis will assess differences between groups for competing time-to-event outcomes (e.g., hospital mortality, time to recovery, time off ventilator, discharge) [68].

Differences in SOFA scores between groups will be evaluated using non-parametric tests. A multinomial ordinal proportional odds model will estimate the odds ratio between study groups for COVID Ordinal Outcomes Scale. For outcomes with repeated assessments, generalized mixed models will evaluate differences between groups with a subject-level random effect to account for repeated measures. The mixed model accounts for data that are missing at random, when including baseline covariates associated with missing status. To account for potential non-ignorable dropout (e.g., due to mortality), a joint modeling approach will incorporate a shared random effect to simultaneously model the time-to-event process and longitudinal process [69].

### Oversight and monitoring

#### Composition of the data monitoring committee, its role and reporting structure

The Ohio State University Center for Clinical and Translational Science (CCTS) established an external DSMB according to NIAID policies. The DSMB is responsible for safeguarding the interests of study participants, assessing the safety and efficacy of study procedures, ensuring data quality, and for monitoring the overall conduct of the study and is independent from the sponsor and any competing interests. The DSMB is asked to make recommendations, as appropriate, to the NIAID about: (1) Efficacy of the study intervention; (2) Benefit/risk ratio of procedures and participant burden; (3) Selection, recruitment, and retention of participants; (4) Adherence to protocol requirements; (5) Completeness, quality, and analysis of measurements; (6) The data and statistical analysis plan; (7) Amendments to the study protocol and consent forms, including whether any new data from other sources affect the equipoise of the study being monitored; (8) Performance of core labs; (9) Participant safety, including review of consent forms; (10) Notification of and referral for abnormal findings; and (11) Participant safety and parent study burden of proposed ancillary studies, including whether the total burden of ancillary studies might compromise the parent study.

The members of the DSMB include independent content experts in pulmonary and critical care medicine, biostatistics, clinical trials, and ethics (some overlapping). The DSMB convened to review the final protocol and DSMB Charter before study initiation and will continue to meet periodically, and not less frequently than annually. The DSMB chairperson will lead each scheduled board meeting and review the prepared board meeting minutes. At each meeting, they will determine whether study progress, data integrity, and safety monitoring warrant continuation of the study and will recommend the study to continue or be terminated, accordingly. The DSMB also approve the data safety plan before the study begins.

It is expected that all DSMB members will attend every meeting and conference call; however, it is recognized that this may not always be possible. A quorum for voting is half of the standing members plus one. A quorum of this DSMB is considered 3 of the 4 standing members, of which the chairperson must be included.

Potential motions of the board meeting include recommendations for either of the following: (1) Study continuation; no change required; (2) Study continuation with stipulation to be formally addressed and approved by the DSMB chair; (3) Study suspension with stipulation to be formally addressed and approved by the DSMB prior to resumption; and (4) Study termination.

The DSMB’s summary report will be reviewed and finalized by the chairperson. The report will then be communicated to the NIAID who will communicate recommendations to the Principal Investigators. DSMB recommendations will be communicated to the IRB in the context of the continuing review process. It is the responsibility of the Principal Investigator to ensure that the IRB is notified by motions requiring termination or suspension of the trial.

An independent Medical Monitor (P.D.) has also been appointed. The Medical Monitor is also independent from the sponsor and any competing interests. Their role is to ensure the safety of trial participants throughout the study and to serve as a point of reference for study team members as they evaluate safety events.

#### Adverse event reporting and harms

The DSMB and Medical Monitor shall receive monthly reports from the PI compiling new and accumulating information on AEs and SAEs recorded on appropriate eCRFs or paper CRFs. In addition, the Medical Monitor shall review and make decisions on the disposition of any SAE received by the PI.

#### Frequency and plans for auditing trial conduct

The DSMB will review all safety data after the first 5 patients and at least yearly thereafter during planned DSMB Data Review Meetings. Data for the planned safety reviews will include, at a minimum, a listing of all reported AEs and SAEs, but access to primary data sources (e.g., the RedCAP database) will also be possible. Such data may be provided blinded by treatment group, with the understanding that unblinding may need to occur in a closed session of the DSMB. In addition, the DSMB will be informed of an Expedited Safety Report in a timely manner (within 14 days).

In addition to the pre‐scheduled data reviews and planned safety monitoring, the DSMB may be called upon for ad hoc reviews. The DSMB will review any event that potentially impacts safety at the request of the PI*.* In addition, the following events will trigger an ad hoc comprehensive DSMB Safety Review: (1) Any death that occurs in the study, which is possibly or definitely related to the study treatment regimen (to be reported within 24 h to DSMB); (2) SAEs that are possibly, probably, or definitely related to the study treatment regimen (reported within 24 h to DSMB); Any common trends in unexpected AEs (reported to DSMB within 5 business days). A temporary halt in study recruitment/randomization will be implemented if an ad hoc DSMB safety review is required. Subjects currently in study and tolerating therapy will be allowed to complete their 5-day course. After review of the data, the DSMB will make recommendations regarding study conduct and/or continuation.

#### Procedure for unblinding

Unblinding must be approved by the study Medical Monitor (P.D.) unless an immediate life-threatening condition has developed, and the Medical Monitor is not accessible. The care team clinician will notify the PI (E.D.C.) and the study statistician of the unblinding event on the next business day. The emergency unblinding will also be reported to the DSMB. A full account of the event will be recorded, including the date and time of the unblinding, the reason for the decision to unblind, and the name of the individual who made the decision and the names of the Medical Monitor and others who were notified. The reasons for unblinding of a participant’s treatment will be included in the final study report.

Any unblinding the study due to an approved interim analysis, final analysis, or study termination will require written approval from NIAID.

### Dissemination plans

All data will be deposited to the Dataverse that is supported by Harvard University under waivers providing for public availability of data repository starting 12 months after the trial begins and will be deposited every 6 months thereafter. This repository provides metadata, persistent unique identifiers, and long-term access for at least 10 years. Data will be findable for the research community under a unique study identifier through the Dataverse repository. All publications will also be deposited in the Dataverse repository. The study will be assigned a digital object identifier (DOI). This data DOI will be referenced in the publication to allow the research community easy access to the exact data used in the publication.

All data will be stored in common and open formats, such as JPEG, CSV, TXT, or PDF. Information needed to make use of this data (e.g., the meaning of variable names, codes, information about missing data, other metadata) along with references to the sources of those standardized names and metadata items will be included wherever applicable. Project-level metadata will be provided at the time of data deposit using Dryad’s web-based deposit form, which conforms to the DataCite metadata schema, a general standard for describing scientific data. No specialized tools, software, and/or code will be needed to access or manipulate shared scientific data.

Data will be made available as soon as possible or at the time of associated publication or end of the performance period. The research community will have access to data at the end of the grant award or when a publication has been submitted. To request access to the data, researchers will use the standard Dataverse processes. The only controls will be those applied by the repository as per the guidelines for authorized access by the public to maintain data safety and integrity.

The researchers’ intention for scientific data management and sharing will be part of the informed consent process. To protect research participants’ privacy and confidentiality, data submitted to the repository will not include personally identifiable information such as names or addresses. Additional protections, such as the approach for managing Health Insurance Portability and Accountability Act identifiers, will be used for de-identification or to provide a limited data set to minimize the risk of participant reidentification. The study datasets will be collected with Health/Medical/Biomedical informed consent. Consequently, the dataset can only be used for studying health, medical, or biomedical conditions and not for the study of population origins or ancestry.

Intermediate and final data from the SCARLET trial will be disseminated via scientific presentations at appropriate local, regional, national, and international Conferences.

The Ohio State University’s policy on the publication of study results will apply to this trial. Intermediate and final study data will also be submitted for publication in appropriate peer-reviewed journals. Biomarker data may also be submitted to appropriate peer-reviewed journals. Eligibility for authorship for all publications will be based on recommended criteria from the International Committee of Medical Journal Editors [70].

If findings from the SCARLET trial are highly significant, The Ohio State University Media Relations Team (a unit of University Communications) will assist with generation of press articles that will facilitate further dissemination of results to the general public. A website may be established to report progress.

### Ethical considerations and compliance with good clinical practice

This clinical study will be conducted using good clinical practice, as delineated in *Guidance for Industry: E6 Good Clinical Practice Consolidated Guidance*, and according to the criteria specified in this study protocol.

As this study is being performed under a research IND, the PI (E.D.C.) is the sponsor. Both the sponsor and the funding agency (the National Institutes of Health) played a role in trial design. The sponsor will oversee and has ultimate authority over collection, management, analysis, and interpretation of data and writing the final study report. The sponsor will work with the co-PI (I.C.D.) to decide where and when to publish the final study report, which will be done regardless of the trial outcome. A steering committee composed of members of the trial team and representatives from NIAID has been established. This committee meets monthly to discuss progress of the trial and any potential concerns or anticipated changes in trial design and conduct.

Before study initiation, the protocol and the informed consent documents were reviewed and approved by the OSU Conflicts Advisory Committee, the OSU Institutional Biosafety Committee, and the OSU Institutional Review Board (IRB). Any amendments to the protocol or to the consent materials will be approved by the IRB before they are implemented.

Each participant’s privacy and confidentiality will be respected throughout the study. Each participant will be assigned a unique identification number and these numbers rather than names will be used to collect, store, and report participant information. Site personnel will not transmit documents containing personal health identifiers to the study sponsors or their representatives.

## Discussion

Citicoline has many characteristics that would be advantageous for any candidate COVID-19 or ARDS therapeutic. Firstly, it has been widely evaluated in both normal and sick human subjects and has an exceptional safety profile. Indeed, food-grade citicoline is classified as GRAS (Generally Regarded as Safe) by the FDA and as safe by the European Food Safety Authority [71]. Citicoline is well tolerated in humans at doses > 1 g/kg/day (below the highest dose proposed for the SCARLET study) and adverse effects are very rare (< 1:10,000) [71–73]. Occasionally, initial digestive intolerance, self-limiting headache, tingling sensation, numbness, and excitability or restlessness have been reported following oral citicoline administration [74–76]. No clinically significant ECG and EEG abnormalities have ever been registered after citicoline administration. Moreover, there is extensive clinical data on citicoline safety. Twenty-four clinical trials of citicoline have been completed to date (see clinicaltrials.gov), primarily for neurologic indications. This includes three trials in which drug was administered i.v.. Frequency of AEs in the largest such trial (ICTUS), which involved 1149 patients on drug, was no higher than in placebo controls [77]. However, citicoline is contra-indicated in patients taking medications that contain L-Dopa, centrophenoxine, or meclofenoxate, and in patients with hypertonia of the parasympathetic nervous system, hence their exclusion from SCARLET.

A large amount of published PK/PD and ADME data is available for citicoline [71, 78]. The pharmacokinetics of oral and intravenous citicoline are almost indistinguishable [79]. In healthy adults, > 99% of a single oral dose of 300 mg of ^14^C-labelled citicoline was absorbed, which resulted in two peaks in plasma radioactivity at 1 h and 24 h post-dose, the second peak being larger [80]. After 5 days, 16% of the administered dose had been recovered, suggesting that the remainder had been incorporated into tissues or was available for biosynthetic and biodegradative pathways. The elimination half-life for CDP-choline that is not incorporated into phospholipids is rapid (56 h for CO_2_ and 71 h for urinary excretion). Hence, it is unlikely to alter the function of cells in which phospholipid synthesis is not impaired [80, 81].

Citicoline is also a simple small molecule with high aqueous solubility and methods for its synthesis are well defined and straightforward [82, 83]. Citicoline is stable in aqueous solution at room temperature for at least 3 years. Hence, it is amenable to pre-pandemic stockpiling and new stocks can be rapidly generated as existing stockpiles become depleted due to expiration or usage. Citicoline’s stability would also facilitate usage in developing countries that lack the resources to maintain cold chains. Moreover, because citicoline targets the host, it is likely to be effective against newly emerging SARS CoV-2 mutants and, unlike vaccines and antivirals, is highly unlikely to constitute a selection pressure for resistance. For the same reason, citicoline will provide a longer window for delayed therapy initiation than current antiviral drugs which are generally only effective early in infection [84, 85].

Importantly for a pulmonary drug, citicoline has excellent bioavailability when administered parenterally [86] so does not need to be instilled directly into the lungs. Hence, issues commonly associated with intra-pulmonary drug administration in ARDS, such as heterogeneity of distribution in the injured lung [87] and induction of transient hypoxemia [88] can be avoided. Moreover, i.v. administration will result in distribution to other affected organs in COVID-19 patients, potentially with beneficial effects.

Finally, citicoline is profoundly anti-inflammatory [58, 89–92]. The apparent beneficial effects of dexamethasone and tocilizumab in COVID-19 [93, 94] suggest that other anti-inflammatory drugs (such as citicoline) may likewise be useful. Given its minimal side effects, citicoline may be superior to corticosteroids, which are harmful in COVID-19 patients not requiring O_2_ [95], increase risk for invasive fungal infections [96–98], and are contra-indicated for other severe respiratory viral infections such as influenza, SARS, and MERS [99].

There are some limitations to the SCARLET study. COVID-19 has a broad spectrum of clinical severity, so designing a pilot trial with statistical power to detect a meaningful difference in ICU-free days or mortality as primary outcome would require an unfeasibly large sample size and could miss significant beneficial effects of citicoline in hospitalized patients. Likewise, the COVID Ordinal Outcomes Scale score [100] would be underpowered to assess efficacy in a small study. As most COVID-19 morbidity relates to HARF and the greatest experimental effect of citicoline is on S_p_O_2_ in mice infected with both IAV [58] and SARS CoV-2 (unpublished observations), we chose to use the lowest S:F ratio value on study day 3 as our primary efficacy readout. P:F and S:F ratios are closely correlated and essentially interchangeable [101–103]. P:F ratios can also be imputed from S_p_O_2_ values [102, 104, 105]. Changes in both are highly and comparably predictive of ICU mortality in ARDS [104]. Lower S:F ratios are also highly predictive of ICU mortality in COVID-19 patients [106, 107].

As a secondary efficacy readout, we will also evaluate the impact of citicoline treatment on SOFA scores in enrolled patients. The SOFA score comprises 0 to 4 points assigned to each of 6 organ systems, based on P:F or S:F ratio, Glasgow Coma Scale score, mean arterial pressure, serum creatinine, serum bilirubin, and platelet count [108]. Higher SOFA scores indicate worse organ function. Although originally designed for sepsis patients, and not universally accepted as being of prognostic value in COVID-19 [109], multiple studies have shown associations between elevated SOFA scores and poor outcomes in this population [110–115]. Moreover, the multisystem nature of the SOFA score may allow us to identify beneficial effects of citicoline on other organs.

Synthesis of CDP-choline is both essential and rate limiting for de novo phosphatidylcholine synthesis and this pathway is highly conserved across all mammals [116]. We propose that ATII cell dysfunction secondary to impaired de novo phospholipid synthesis is a central player in the pathogenesis of ARDS from multiple causes, including infection other RNA viruses with pandemic potential, such as IAV and other coronaviruses (SARS and MERS). We also propose that this host ATII cell dysfunction is readily reversed by treatment with citicoline. Given its broad efficacy, safety, favorable chemical characteristics, and potentially pathogen-agnostic efficacy, successful demonstration that citicoline is beneficial in severely ill patients with SARS CoV-2-induced HARF could transform management of severely ill COVID patients.

## Trial status

Protocol version: 4.81, December 5th, 2023.

Date of first enrollment: 14th July, 2023.

Approximate date for completion of recruitment: March 2025.

### Supplementary Information

Supplementary file 1. The Ohio State University Combined Consent to Participate in Research and HIPAA Research Authorization.

## Data Availability

Our ethical and legal responsibility to respect participants’ rights to privacy and to protect their identity will inform our policy for sharing clinical data once the trial has been completed. We will gain informed consent for publication of the dataset from participants at the point of enrollment in the trial. Clinical datasets will not contain any direct or indirect identifiers and participants will remain fully anonymous.

## Abbreviations

AE: Adverse event
ARDS: Acute respiratory distress syndrome
ATII cell: Alveolar type II respiratory epithelial cell
CCT-α: CTP-phosphocholine cytidylyltransferase-α
CDP-choline: Cytidine 5’diphosphocholine
COVID-19: Coronavirus disease 2019
DSMB: Data Safety Monitoring Board
eCRF: Electronic case report form
EMR: Electronic medical record
F_i_O_2_: Fraction of inspired oxygen, %
HARF: Hypoxemic acute respiratory failure
ICU: Intensive care unit
IDS: Investigational Drug Service
IRB: Institutional Review Board
LAR: Legally authorized representative
OSU: The Ohio State University
P_a_O_2_: Arterial partial pressure of oxygen (index of hypoxemia), mmHg
P:F: P_a_O_2_:F_i_O_2_ ratio, mmHg
REDCap: Research Electronic Data Capture
SAE: Serious adverse event
SAR: Serious adverse reaction
SARS CoV-2: Severe acute respiratory syndrome coronavirus-2
SCARLET: Supplemental Citicoline Administration for Resolution of Lung injury Efficacy Trial
S:F: S_p_O_2_:F_i_O_2_ ratio
SOFA: Sequential Organ Failure Assessment
S_p_O_2_: Peripheral oxygen saturation (%)
WMC: Wexner Medical Center
