# Supplementary material for: SCARLET (Supplemental Citicoline Administration to Reduce Lung injury Efficacy Trial): study protocol for a single-site, double-blinded, placebo-controlled, and randomized Phase 1/2 trial of i.v. citicoline (CDP-choline) in hospitalized SARS CoV-2-infected patients with hypoxemic acute respiratory failure
Source: Trials. 2024 May 18;25:328. doi: 10.1186/s13063-024-08155-0 (PMC11102211; doi:10.1186/s13063-024-08155-0)
Supplement: Supplementary file 1 — The Ohio State University Combined Consent to Participate in Research and HIPAA Research Authorization. [file 13063_2024_8155_MOESM1_ESM.docx]

Additional file 1: The Ohio State University Combined Consent to Participate in Research and HIPAA Research Authorization

| **Study Title:** | **SCARLET**” (**S**upplemental **C**iticoline **A**dministration to **R**educe **L**ung injury **E**fficacy **T**rial) |
| --- | --- |
| **Principal Investigator:** | Elliott Crouser, MD |
| **Sponsor:** | National Institute of Allergy and Infectious Diseases |

- **This is a consent form for research participation.** It contains important information about this study and what to expect if you decide to participate. Please consider the information carefully. Feel free to discuss the study with your friends and family and to ask questions before making your decision whether or not to participate.
- **Your participation is voluntary.** You may refuse to participate in this study. If you decide to take part in the study, you may leave the study at any time. No matter what decision you make, there will be no penalty to you, and you will not lose any of your usual benefits. Your decision will not affect your future relationship with The Ohio State University. If you are a student or employee at The Ohio State University, your decision will not affect your grades or employment status.
- **You may or may not benefit as a result of participating in this study.** Also, as explained below, your participation may result in unintended or harmful effects for you that may be minor or may be serious depending on the nature of the research.
- **You will be provided with any new information that develops during the study that may affect your decision whether or not to continue to participate.** If you decide to participate, you will be asked to sign this form and will receive a copy of the form. You are being asked to consider participating in this study for the reasons explained below.

1. **Why is this study being done?**

You are being asked to take part in this study because you have been admitted to the hospital with COVID-19, which is caused by a virus named SARS CoV-2. This viral infection may cause inflammation that damages the small air sacs in your lung, called alveoli. When the air sacs are damaged this causes difficulty getting oxygen from your lungs into your blood stream. To support your body during this time you doctors have placed you on additional oxygen either via a nasal cannula (oxygen tube), mask, or ventilator. In addition, your doctors may be treating you with a variety of medications to either help treat the virus, antiviral medications, and help with any inflammation in your lungs.

One of the ways that the SARS CoV-2 virus damages the lung is by reducing the lungs’ ability to produce a natural compound, called surfactant, which acts to help keep the lungs open and protects those small air sacs. Loss of surfactant means it is harder to breath and harder to get oxygen into your blood stream.

In animal experiments, a medication, **citicoline,** which is a building block of surfactant, was able to restore the lungs’ function and improve oxygenation in mice infected with SARS CoV-2 virus. The purpose of this study is to see if this medication, citicoline, can be used safely in patients with COVID-19 and to find out if it helps with their oxygen levels.

**2. How many people will take part in this study?**

A total of 80 patients will take part in this study at The Ohio State University.

**3. What will happen if I take part in this study?**

**Medication Dosing**

Once you have signed this consent, you will be randomized to receive either one of three different doses of citicoline or a placebo (saline) every 12 h for 5 days. This will be done in a blinded fashion so that neither your doctor or you will know which medication and dose you are getting.

**Blood Collection**:

We will collect at least 1-2 teaspoons of blood on days 1–5 of the study. We will also collect blood on day 8 of the study if you remain hospitalized at that time. If your treating doctors already ordered blood tests for you, we will use those if possible. Your nurse will attempt to use an existing IV to draw your blood to avoid any pain. Should having your blood drawn become painful, you may tell us to stop at any time.

One of our research coordinators will also look at your medical record and copy down some information about your medical history, why you were admitted to the hospital, and how you are doing every day while you are admitted. We will copy down the results of some of the laboratory studies that your doctors are ordering for you as well.

All samples will be coded with a study number, and we will try very hard to protect your privacy. Only the study team will know your study number, which will be kept in a locked file. The stored samples will not contain your name or identifying information, but we can link samples to your name using the study number, if need be.

**Mini-BAL**

If you are on a ventilator, we will attempt to take samples of the immune cells from your lung on days 1, 3, and 5 of the study. We will also collect a sample on day 8 if you remain on a ventilator at this time. This procedure will only be done if your doctors and treating team feel it is safe. This will be similar to the suctioning through the breathing tube that you may have already seen your care providers use to clear your throat. Basically, a slightly longer catheter will be put in your breathing tube and about 6 teaspoons of saline will be injected into the catheter. We will then suck out this saline and analyze the fluid and cells from your lung that come with it. This may cause some coughing. This will only be done while you are on the ventilator: if you are not or no longer on a ventilator, no sample will be collected. You may refuse this procedure and still be in the study.

**Follow-Up**:

After you leave the hospital, one of our research coordinators will contact you by telephone before the end of the first month to enquire about your health. Once this information has been gathered, you will not be contacted again.

Many of our patients are interested in hearing about any results from our study. This study will not generate any information about you personally, but we would be happy to send you updates on the study and any discoveries made if you include a contact email at the bottom of this form. Including this email is totally voluntary and does not impact your participation in the study.

**4. How long will I be in the study?**

We will collect blood from you while you are getting the study drug and on day 8 after your first dose. We will keep checking your records during your hospital stay until you are discharged from the hospital. There will be no long-term follow-up needed on your part.

# Can I stop being in the study?

You may leave the study at any time. If you decide to stop participating in the study, there will be no penalty to you, and you will not lose any benefits to which you are otherwise entitled. Your decision will not affect your future relationship with The Ohio State University.

1. ***What risks, side effects or discomforts can I expect from being in the study?***

We will do everything possible to minimize the risks of you being in this study.

**Risk of Study Drug**

Citicoline is generally felt to be safe. It has been used as a drug and nutritional supplement in Europe and Japan. Patients taking citicoline by mouth have reported generally mild side effects including: trouble sleeping, headache, constipation, diarrhea, nausea, stomach pain, blurred vision, and chest pains. We do not know all the side effects that may occur with citicoline in a severely ill patient. We will be monitoring you for side effects while you are in the study. Your treating doctor can stop treatment at any time if they feel that you have any significant side effects.

**Risk of Blood Draw**

This study will require us to draw you blood which can be painful. We will attempt to minimize this by asking your nurse to draw your blood. If you have pain with a blood draw you may ask us to stop at any time. Drawing blood may also result in minor bleeding, bruising, or swelling of the collection site.

**Risk of “mini-bronchoalveolar lavage”**

This will only be done for patients on ventilator. The suctioning of saline from your trachea will likely cause coughing while the catheter is in place. The cough usually improves shortly after the procedure. There is a small risk that your oxygen level may drop after procedure and you would temporarily need increase in your oxygen concentration on the ventilator. There is a small risk that the catheter could cause irritation of your trachea resulting in bleeding. To prevent significant bleeding, we will only do this procedure if your blood work shows you are not at high risk for bleeding and if your treating doctor feels it is safe.

**Loss of confidentiality**

We will be collecting your information from your medical chart. This will include some identifiable information such as your name and age and OSU medical record number. There is a potential that this information could be stolen. To minimize this, we will only store this information on an OSU password protected server. It will not be stored on any portable storage device that could be lost or stolen.

1. **What benefits can I expect from being in the study?**

You may receive the drug, citicoline. We do not know if this study drug is beneficial or helps with your condition. There is no benefit to you for taking part in this study other than helping understand how we may be better able to treat patients such as yourself in the future.

**8. What other choices do I have if I do not take part in the study?**

This study will not impact your medical care in any way. You may choose not to participate without penalty or loss of benefits to which you are otherwise entitled. Should you decline to participate in this study, your ICU team will continue to offer whatever treatment they feel is best for you.

**9. What are the costs of taking part in this study?**

You will not be charged for any study activities.

**10. Will I be paid for taking part in this study?**

You will not be paid for being in this study.

11. What happens if I am injured because I took part in this study?

In the unlikely event you suffer an injury from participating in this study, you should notify the researcher or study doctor immediately, who will determine if you should obtain medical treatment at The Ohio State University Wexner Medical Center.

The cost for this treatment will be billed to you or your medical or hospital insurance. The Ohio State University has no funds set aside for the payment of health care expenses for this study.

# 12. What are my rights if I take part in this study?

If you choose to participate in the study, you may discontinue participation at any time without penalty or loss of benefits. By signing this form, you do not give up any personal legal rights you may have as a participant in this study.

You will be provided with any new information that develops during the course of the research that may affect your decision whether or not to continue participation in the study.

You may refuse to participate in this study without penalty or loss of benefits to which you are otherwise entitled.

An Institutional Review Board responsible for human subjects research at The Ohio State University reviewed this research project and found it to be acceptable, according to applicable state and federal regulations and University policies designed to protect the rights and welfare of participants in research.

# 13. Will my study-related information be kept confidential?

The NIH issues Certificates of Confidentiality for all NIH-funded studies, including this study. This Certificate provides extra protection for you and your study information, documents, or samples (blood, tissue, etc.). The Certificates are issued so that we cannot be required to disclose any identifiable information collected about you as a part of this study in a lawsuit or legal proceeding. This is a layer of protection over and above the already existing protections in place for you and your information, documents, or samples.

Efforts will be made to keep your study-related information confidential. However, there may be circumstances where this information must be released. For example, personal information regarding your participation in this study may be disclosed if required by state law.

Also, your records may be reviewed by the following groups (as applicable to the research):

- Office for Human Research Protections or other federal, state, or international regulatory agencies;
- U.S. Food and Drug Administration;
- The Ohio State University Institutional Review Board or Office of Responsible Research Practices;
- The sponsor supporting the study, their agents or study monitors; and
- Your insurance company (if charges are billed to insurance).

# 14. HIPAA AUTHORIZATION TO USE AND DISCLOSE INFORMATION FOR

# RESEARCH PURPOSES

1. **What information may be used and given to others?**

- Past and present medical records;
- Research records;
- Records about phone calls made as part of this research;
- Records about your study visits;
- Information that includes personal identifiers, such as your name, or a number associated with you as an individual;
- Information gathered for this research about:

Physical exams

Laboratory, x-ray, and other test results

1. **Who may use and give out information about you?**

Researchers and study staff.

1. **Who might get this information?**

- The sponsor of this research. “Sponsor” means any persons or companies that are:
  - working for or with the sponsor; or
  - owned by the sponsor.
- Authorized Ohio State University staff not involved in the study may be aware that you are participating in a research study and have access to your information;
- If this study is related to your medical care, your study-related information may be placed in your permanent hospital, clinic or physician’s office record;
- Others: The National Institute of Allergy and Infectious Diseases

1. **Your information may be given to:**
2. The U.S. Food and Drug Administration (FDA), Department of Health and Human Services (DHHS) agencies, and other federal and state entities;
3. Governmental agencies in other countries;
4. Governmental agencies to whom certain diseases (reportable diseases) must be reported; and
5. The Ohio State University units involved in managing and approving the research study including the Office of Research and the Office of Responsible Research Practices.
6. **Why will this information be used and/or given to others?**

- To do the research;
- To study the results; and
- To make sure that the research was done right.

1. **When will my permission end?**

There is no date at which your permission ends. Your information will be used indefinitely. This is because the information used and created during the study may be analyzed for many years, and it is not possible to know when this will be complete.

1. **May I withdraw or revoke (cancel) my permission?**

Yes. Your authorization will be good for the time period indicated above unless you change your mind and revoke it in writing. You may withdraw or take away your permission to use and disclose your health information at any time. You do this by sending written notice to the researchers. If you withdraw your permission, you will not be able to stay in this study. When you withdraw your permission, no new health information identifying you will be gathered after that date. Information that has already been gathered may still be used and given to others.

1. **What if I decide not to give permission to use and give out my health information?**

Then you will not be able to be in this research study and receive research-related treatment. However, if you are being treated as a patient here, you will still be able to receive care.

1. **Is my health information protected after it has been given to others?**

There is a risk that your information will be given to others without your permission. Any information that is shared may no longer be protected by federal privacy rules.

1. **May I review or copy my information?**

Signing this authorization also means that you may not be able to see or copy your study-related information until the study is completed.

# 15. Who can answer my questions about the study?

For questions, concerns, or complaints about the study, or if you feel you have been harmed as a result of study participation, you may contact Dr. Matthew Exline at 614-293-4925.

For questions related to your privacy rights under HIPAA or related to this research authorization, please contact the HIPAA Privacy Officer at 614-292-2856, Meiling Hall Room 271 370 W. 9^th^ Ave., Columbus, OH 43210.

For questions about your rights as a participant in this study or to discuss other study-related concerns or complaints with someone who is not part of the research team, you may contact Ms. Sandra Meadows in the Office of Responsible Research Practices at 1-800-678-6251.

If you are injured as a result of participating in this study or for questions about a study-related injury, you may contact Dr. Elliott Crouser at 614-293-4925.

**CONSENT FOR FOLLOW-UP INFORMATION ON STUDY**

By providing your email, you are consenting for us to contact you via email with any results, publications, or grants that result from this study. This information will be on the study as a whole and will not include any of your personal information or personal study results. You may stop these emails at any time by responding to take you off our mailing list. You will not be asked for any additional information. Your email will not be shared with any other organizations or researchers. This option is purely for your interest and does not impact your participation in the study in any other way. Sharing email is completely voluntary.

Primary Email:____________________________________________________________

Secondary Email:__________________________________________________________

# Signing the consent form

I have read (or someone has read to me) this form and I am aware that I am being asked to participate in a research study. I have had the opportunity to ask questions and have had them answered to my satisfaction. I voluntarily agree to participate in this study.

I am not giving up any legal rights by signing this form. I will be given a copy of this combined consent and HIPAA research authorization form**.**

|  |  |  | |
| --- | --- | --- | --- |
| **Printed name of subject** |  | **Signature of subject** | |
|  |  |  | **AM/PM** |
|  |  | **Date and time** |  |
|  |  |  |  |
|  |  |  | |
| **Printed name of person authorized to consent for subject (when applicable)** |  | **Signature of person authorized to consent for subject**  **(when applicable)** | |
|  |  |  | **AM/PM** |
| **Relationship to the subject** |  | **Date and time** |  |

**Investigator/Research Staff**

I have explained the research to the participant or his/her representative before requesting the signature(s) above. There are no blanks in this document. A copy of this form has been given to the participant or his/her representative.

|  |  |  | |
| --- | --- | --- | --- |
| **Printed name of person obtaining consent** |  | **Signature of person obtaining consent** | |
|  |  |  | **AM/PM** |
|  |  | **Date and time** |  |

**Witness(es) -** *May be left blank if not required by the IRB*

|  |  |  | |
| --- | --- | --- | --- |
| **Printed name of witness** |  | **Signature of witness** | |
|  |  |  | **AM/PM** |
|  |  | **Date and time** |  |
|  |  |  | |
| **Printed name of witness** |  | **Signature of witness** |  |
|  |  |  | **AM/PM** |
|  |  | **Date and time** |  |

**Laboratory Plan.**

All OSU hospital clinical laboratories are Clinical Laboratory Improvement Amendment (CLIA)-approved by the Dept. of Health and Human Services and are accredited by the College of American Pathologists (CAP)’s Laboratory Accreditation Program. All clinical laboratory testing for study participants will comply with 42 CFR part 493.2 and 493.3(b)(2).

**Samples collected.** The following patient sample types will be collected, processed, and analyzed:

1. Venous blood - drawn at Time 0 and then once daily on study days 1–5 and 8 (all patients)
2. Venous blood – drawn at Time 0, 1, 2, 4, 8, and 12 h after administration of the first citicoline bolus for pharmacokinetic analysis (6 patients per group)
3. Bronchoalveolar lavage fluid – sampled on study days 1, 3, 5, and 8 (all patients)

All patient samples will initially be processed by a research coordinator in the clinical wet labs available at OSU East and OSU Main. All patient samples will be labeled with date and time of acquisition and patient study ID number and will remain blinded to the study team.

**Processing of venous blood samples.** At each study timepoint, a sufficient volume of venous blood will be drawn into blood container tubes containing appropriate anticoagulants to complete all the following assays:

1. CBC/differential count
2. Hematology panel
3. Renal function clinical chemistry panel
4. Cardiac function clinical chemistry panel
5. Hepatic function clinical chemistry panel
6. PT & PTT time
7. D-dimer, ferritin, fibrinogen
8. Thromboelastography

These assays will be performed by the clinical lab at the hospital in which that subject is located.

An additional 10 ml heparinized venous blood will be collected from each subject at each study timepoint for research purposes. If collected at OSU East, blood samples will be transferred to OSU Main via the hourly shuttle service before further processing. Venous blood collected for research use from patients at both OSU East and OSU Main will be transferred to Dr. Crouser’s laboratory in the Davis Heart and Lung Research Institute (part of OSU Main). Dr. Crouser’s research associate, Mr. Mark Julian, will separate plasma and leukocytes. Plasma will be divided into 1 ml aliquots and stored in a locked -80°C freezer for batch analysis of biomarkers. Leukocytes will be resuspended in freezing media, aliquoted into cryovials, and stored at -80°C for downstream research studies.

**Processing of mini-BAL samples.** The volume of BAL fluid recovered is recorded and the lavagate is filtered through a double layer of sterile gauze swab to remove mucus plugs into prechilled, sterile 50 ml centrifuge tubes. Cells are pelleted by centrifugation at 500 x g at 4 °C for 5-10 min and the supernatant will be collected and frozen in 1 ml aliquots, as for plasma. The cell pellet will then be washed by vortexing in 50 ml cold normal saline. Centrifugation is repeated once. Cells are then resuspended in RPMI-1640 (+ 10% fetal bovine serum + 2 mM L-glutamine + penicillin [40 IU/ml], streptomycin [75 IU/ml], and amphotericin B [0.5 IU/ml]) and differential counting performed using an equal volume of trypan blue and a hemocytometer. Cells will again be pelleted, resuspended in freezing media at a concentration of 1 x 10^6^ cells/ml, aliquoted into cryovials at 1 x 10^6^ cells/vial, and stored at -80°C for downstream research studies.

**Biomarker analysis.** Once sufficient plasma and BALF samples have been collected to facilitate batch analysis (40), aliquots of both will be transferred to a locked -80°C freezer in the Davis lab. Effects of citicoline on a variety of potential biomarkers of ARDS and/or COVID-19 severity will be determined.

1. IL-6, IL-8, IL-10, IL-18, and other cytokines and chemokines in plasma and BALF will be measured by Bio-Plex (48-Plex Human Cytokine Screening Panel).
2. Plasma and BALF lactate dehydrogenase, RAGE, and SP-D will be assayed using commercial ELISA kits.
3. Plasma IFN-a, IFN-l, Angiopoietin 2, and C-reactive protein will be assayed using commercial ELISA kits.
4. Plasma viremia will be quantified by qRT-PCR for SARS CoV-2 N gene using the CDC protocol.

Batched assays will be performed in accordance with Good Laboratory Practice principles using validated commercial reagents.

**Measurement of plasma citicoline levels.** Plasma samples collected for citicoline analysis will be transferred to a locked -80°C freezer in the Davis lab. They will be transferred in batches to Dr. Riedl for analysis of citicoline, CDP, and choline levels by HPLC. Unused samples will be returned to Dr. Davis. HPLC analyses will use HPLC-grade buffers, solvents, and other reagents.
